# Inertial Motion Capture-Based Wearable Systems for Estimation of Joint Kinetics: A Systematic Review

**DOI:** 10.3390/s22072507

**Published:** 2022-03-25

**Authors:** Chang June Lee, Jung Keun Lee

**Affiliations:** 1 Department of Mechanical Engineering, Hankyong National University, Anseong 17579, Korea; 2019563205@hknu.ac.kr; 2 School of ICT, Robotics & Mechanical Engineering, Hankyong National University, Anseong 17579, Korea

**Keywords:** inertial motion capture, joint kinetics, wearable system, inverse dynamics, machine learning

## Abstract

In biomechanics, joint kinetics has an important role in evaluating the mechanical load of the joint and understanding its motor function. Although an optical motion capture (OMC) system has mainly been used to evaluate joint kinetics in combination with force plates, inertial motion capture (IMC) systems have recently been emerging in joint kinetic analysis due to their wearability and ubiquitous measurement capability. In this regard, numerous studies have been conducted to estimate joint kinetics using IMC-based wearable systems. However, these have not been comprehensively addressed yet. Thus, the aim of this review is to explore the methodology of the current studies on estimating joint kinetic variables by means of an IMC system. From a systematic search of the literature, 48 studies were selected. This paper summarizes the content of the selected literature in terms of the (i) study characteristics, (ii) methodologies, and (iii) study results. The estimation methods of the selected studies are categorized into two types: the inverse dynamics-based method and the machine learning-based method. While these two methods presented different characteristics in estimating the kinetic variables, it was demonstrated in the literature that both methods could be applied with good performance for the kinetic analysis of joints in different daily activities.

## 1. Introduction

Joint kinetics, which considers the force, moment, and power exerted on joints, has an important role in assessing human motor functions and diagnosing diseases in different applications, such as rehabilitation, medical care, and sports. For example, knee adduction moments, which are related to the medial–lateral (ML) load at the knee joint, are frequently used as quantitative indicators to assess the severity and progression of knee osteoarthritis (OA) [1,2,3,4]. In addition, to assist walking through a lower limb exoskeleton robot, the joint moments of the lower body must be measured [5,6].

An approach commonly used for joint kinetic analysis is inverse dynamics, where Newton–Euler equations are sequentially applied to a series of articulated body segments to calculate the forces and moments applied to each joint [7,8,9,10]. The joint power is calculated using the dot product of the joint moment and the angular velocity. There are three types of input data used in performing inverse dynamics: (i) the kinematic data of segments and joints, (ii) the external forces exerted on the segments, and (iii) the body segment inertial parameters. Kinematic data include segment orientation and position, linear/rotational velocity and acceleration, and joint position and axis, which are measured using an optical motion capture (OMC) system that tracks optical markers attached to the body with multiple infrared cameras. For example, the three-dimensional (3D) orientation and origin of anatomical coordinate systems for body segments can be defined from optical markers attached to anatomical indicators based on the recommendations of the International Society of Biomechanics [11,12]. External force information includes the ground reaction force (GRF), moment (GRM), and center of pressure (CoP), which can be measured using a force plate (FP). Finally, body segment inertial parameters include the segment mass, center of mass (CoM) position, and inertial moment of each body segment, which are predetermined based on the subject’s body weight and height [13,14,15]. Therefore, kinetics analysis is typically performed using an OMC system in combination with FPs.

However, the OMC system has a problem in that the range of activities that can be measured is limited because it only operates where the equipment is preinstalled (often known as the in-the-lab limitation). Furthermore, this system is expensive in general. To overcome these limitations of the conventional system, numerous recent studies have utilized low-cost and lightweight wearable sensing technologies. An inertial measurement unit (IMU) is a sensor module where an accelerometer, a gyroscope, and a magnetometer measure linear acceleration (including gravitational acceleration), rotational angular velocity, and global magnetic fields, respectively. In various applications, the IMUs are used to detect human motion [16,17,18,19] or estimate the 3D orientation information of an object by combining sensor signals within the fusion algorithm [20,21,22,23]. The inertial motion capture (IMC) system, which tracks human motion based on IMU technology, has the potential to replace the conventional OMC systems that require fixed cameras and multiple markers. Accordingly, numerous studies have used the IMC system to kinematically analyze human motions. Moreover, it is also easy to find review papers that assess the kinematics of specific joints, such as the upper limb [24], lower limb [25], shoulder [26], and spine [27].

Furthermore, studies are being conducted to estimate joint kinetics and kinematic information through the IMC system. There are relatively fewer published papers on joint kinetics compared to joint kinematic studies. However, they must be examined comprehensively as the importance of joint kinetics is exponentially growing. Recently, Ancillao et al. [28] published a review paper on the IMC system-based estimation of kinetic quantities, focusing on methodologies for estimating GRF, GRM, and CoP using IMU data. However, the majority of the studies discussed in [28] were not extended to joint kinetics. Adesida et al. [29] reviewed papers evaluating sports activities in terms of kinematics and kinetics using wearable sensors, including IMUs. However, these did not include papers evaluated on joint forces or moments. Gurchiek et al. [30] reviewed papers estimating biomechanical physical quantities using wearable sensors based on machine learning techniques. However, the studies that estimated joint moments used surface electromyography sensors rather than IMUs. Hence, to date, research on estimating and analyzing the kinetic information of joints based on IMC systems has not been comprehensively addressed. 

The purpose of this review is to examine the methodology of the current studies on estimating joint kinetic variables by means of an IMC-based wearable system. Specifically, in this paper, we summarized the content of the literature in terms of the study characteristics, methodologies, and study results.

## 2. Methods

### 2.1. Search Strategy

This review was conducted in accordance with the preferred reporting items for systematic reviews and meta-analyses (PRISMA) statement [31]. The PubMed and IEEE Xplore databases were searched for relevant papers in August 2021. The search terms were selected to reflect three main categories: joint, kinetics, and IMU. Table 1 lists the search terms applied for the literature search in this review. “OR” was used to include at least one of the words corresponding to each keyword. “AND” was used to include all three keywords. After removing the duplication, the title and abstract of each paper were evaluated to select papers according to eligibility criteria. 

### 2.2. Inclusion/Exclusion Criteria

In this review, we evaluated papers that contained detailed descriptions of the developed system or methodology for estimating joint kinetic variables. The inclusion criteria were as follows: journal or conference papers written in English; research on estimating kinetic variables including joint force, moment, and power using IMUs alone (including either accelerometers or gyroscopes) or IMUs in combination with additional sensors such as force plates and pressure sensors; and papers that described the sensor configuration, the method for estimating the joint kinetic variables, and the experimental process (experimental setup, motion, and participating subject). Moreover, studies that compared several methods for estimating the joint kinetics were included. If a journal paper and a conference paper by the same author contained similar content, the journal paper was included first. Papers that met the following criteria were excluded: studies concentrating on estimating kinematic variables, GRF, and muscle strength, and papers that did not sufficiently describe a method or system.

### 2.3. Data Extraction

Information was extracted from the selected papers and is summarized in Table 2, Table 3, Table 4 and Table 5. The extracted information included the characteristics of the study (Table 2), the methodologies (Table 3 and Table 4), and the study results (Table 5). The characteristics of the study included the analyzed activities and joints, the kinetic variables, the types of estimation methods, sensor configuration, and information regarding the subjects who participated in the experiment. The estimation methods were categorized into an inverse dynamics-based method (IDM) and a machine learning-based method (MLM). The characteristics of the former included the location and number of IMUs attached, the sensor or prediction method for measuring an external force, the method for estimating the joint kinetics, and the assumptions or features. The latter included the location and number of IMUs attached, the machine learning technique applied to train the model, and the input data. Lastly, the study results include the outcomes and evaluation metric.

## 3. Results

**Figure 1:**
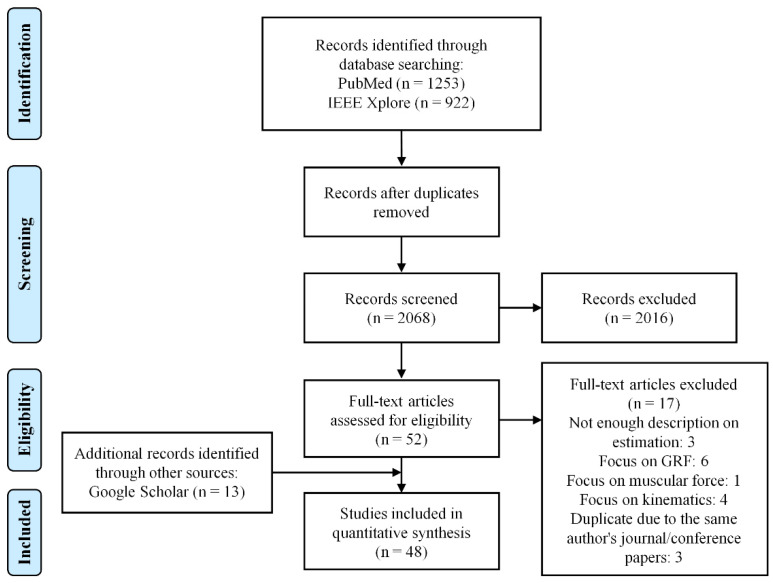
PRISMA flow diagram.

From a systematic review, 2175 papers (1253 from PubMed and 922 from IEEE Xplore) were identified, and after removing 107 duplicates, 52 papers were selected by screening the titles and abstracts of 2068 papers. After reading the full text of the 52 selected papers, 17 papers were excluded for the following reasons: not enough description of the estimation method, focus on the estimation of GRF, muscular force, and kinematics and duplicate content due to the same author’s journal and conference papers. After that, 48 papers were finally selected by adding 13 papers identified from other sources (Google Scholar). The literature search, selection, and data extraction were performed by both authors. Figure 1 shows the PRISMA flow chart describing the selection procedure of the papers for a systematic review. 

The content of the selected papers was largely categorized into the characteristics of the studies, methodologies, and study results. These are discussed in Section 3.1, Section 3.2 and Section 3.3, respectively. Abbreviations lists the definitions of the abbreviations frequently used in this review.

### 3.1. Characteristics of Studies

Table 2 presents the activities and joints to be analyzed, the kinetic variable, the type of method, the measurement system, and the subject information.

#### 3.1.1. Activity

The selected studies estimated the kinetic variables of the joints by targeting different motions or sports activities that were closely related to daily life. Among these, the most evaluated activity was walking, which is a fundamental human movement. In 29 of the 46 selected papers, level walking was included in the experimental motions [32,33,34,35,36,37,38,39,40,41,42,43,44,45,46,47,48,49,50,51,52,53,54,55,56,57,58,59,60], and 23 of these discussed only walking [32,33,34,35,36,37,38,39,40,41,42,43,44,45,46,47,48,49,50,51,52,53,54]. While most of these studies performed straight walking, two studies performed walking turns in addition to walking straight [57,60]. Four studies [56,57,59,60] performed running straight. Among them, two studies additionally performed running turns [57,60]. Other activities analyzed included lifting and carrying loads [61,62,63,64,65,66,67], sit-to-stand [55,58,68,69,70,71], stair ascent and descent [55,58,70,72], standing [58,73,74], and trunk bending [75]. In addition, four studies conducted a kinetic analysis of winter sports, including snowboarding [76], ski jumping, [77], skiing [78], and skating [79].

#### 3.1.2. Joint under Study

The majority of the studies focused on the kinetics of the hip, knee, and ankle joints, as they are the major joints of the lower body. Among them, 21 studies analyzed the physical quantities of all three joints, and three, seven, and six papers analyzed only one of the hip, knee, and ankle joints, respectively (see Table 2). In ten studies, the loading of the lumbar joint (e.g., L5/S1 joint) was estimated [40,61,62,63,64,65,66,67,74,75]. Kim and Nussbaum [62] estimated the moment for the shoulder joint, lower body, and L5/S1 joints. Larsen et al. [66] estimated the force applied to the L4-L5 discs and the force of the erector spinae muscle based on the musculoskeletal model. Khurelbaatar et al. [40] estimated the forces and moments of nine joints located in the upper and lower bodies and the torso.

#### 3.1.3. Kinetic Variable

In the majority of the studies, the joint moment indicating the mechanical load experienced by the joint structures was estimated as a kinetic variable to evaluate the motor function of the joint (38 studies). Of these, all three axial components were evaluated in 19 studies, and the ML axial component (i.e., flexion moment) and anterior–posterior (AP) axial component (i.e., adduction moment) were evaluated in 14 and 4 studies, respectively. Khurelbaatar et al. [40] evaluated the magnitude of the moment. In 15 studies, the reaction force of the joint was estimated, and in [49], the tibial compression force, which was the sum of the ankle reaction force and muscular force, was estimated. De Brabandere et al. [58] estimated the impulse obtained by time-integrating the contact force to evaluate the joint load that occurs when performing a one-movement cycle. In addition, five studies estimated joint power.

#### 3.1.4. Estimation Method and Measurement System

The methods for estimating the joint kinetic variables in the selected papers can be categorized into the following two types: (i) the inverse dynamics-based method (IDM) and (ii) the machine learning-based method (MLM). The former is a method that performs inverse dynamics based on the kinematics of human bodies measured by an IMC system to estimate the joint kinetics. The external force required for inverse dynamics is measured using additional force sensors, such as FP, mobile force plate (MFP), or pressure sensor (PS), or is predicted from the IMU data. The latter is a method that trains a model to estimate the joint kinetic variables from the IMU data using machine learning techniques. Both types of method are described in detail in Section 3.2.

#### 3.1.5. Subjects

In all the selected studies, experiments were conducted on subjects of different ages and genders, and in 25 studies, more than ten subjects participated in the experiment. In the majority of the studies, kinetic analysis was performed on healthy subjects, and only seven studies conducted experiments on patients. Six of these studies included patients with OA of the hip (one study), knee (three studies), and ankle (two studies). The other included patients with limb dyskinesia. In addition, three of these studies compared and analyzed the results of two groups of subjects, patients, and healthy adults. In the other six studies, athletes or sports students participated in the evaluation of the joint load during sports activities.

### 3.2. Methodologies

This section examines the methods for estimating the joint kinetic variables in the selected papers. As described above, the estimation methods are divided into two types (i.e., IDM and MLM) which have different processes and characteristics for estimating joint kinetics. Thus, IDM and MLM are described in Section 3.2.1 and Section 3.2.2, respectively, for an effective comparative analysis. Table 3 and Table 4 summarize the descriptions of IDM and MLM, respectively.

#### 3.2.1. Inverse Dynamics-Based Method (IDM)

##### Sensor Attachment Location

In studies estimating joint kinetics using IDM, the kinematics of the lower body or entire body were estimated by means of IMUs attached to body segments based on a biomechanical model to perform inverse dynamics. As the majority of the studies focused on the joint kinetics of the lower extremities, the IMUs were attached mainly to lower body segments, including the pelvis, thigh, shank, and foot. The IMUs were also attached to the chest and upper body segments. For example, Zijlstra and Bisseling [73] attached sensors to the chest and pelvis. In 11 studies, full-body kinematics were measured using Xsens MVN Link (Xsens Technologies BV, Enschede, The Netherlands), where the IMUs were attached to 17 segments of the upper body, lower body, and trunk. In this case, the measured data were processed using the dedicated software Xsens MVN Studio, which provides an anatomical model.

##### Dimension

Twelve studies considered only two-dimensional (2D) movements, assuming that the human body motion is bilaterally symmetrical or that the segments move in a single plane and express the posture of the segment as a single angle. In this case, the 2D force on the sagittal plane and moment of the ML axis (i.e., flexion moment) were estimated. As an exception, Zijlstra and Bisseling [73] considered only motion in the frontal plane to estimate the adduction moment of the hip joint. In studies other than the above literature, the forces and moments of the joints about three axes were estimated by performing inverse dynamics based on a 3D biomechanical model.

##### Anatomical Calibration

Because the coordinate system of the sensor attached to the body segment and the anatomical coordinate system of the segment are different, a calibration procedure is required to align these two coordinate systems. In six papers, specific axes with respect to the sensor coordinate system were found by using sensor signals during specific static postures or motions [32,36,56,64,74,77]. In three of these papers [32,36,74], the flexion/extension of the knee or hip was performed to find the ML axis of the joint based on the angular velocity of the gyroscope signal, while the static upright posture was used to find the superior–inferior (SI) axis based on the gravitational acceleration of the accelerometer signal. Then, the third axis could be determined based on the cross product of the two axes. Dorschky et al. [56] performed different movements, including standing upright and bending the trunk, leg, and foot in the sagittal plane. Conforti et al. [64] measured the gravitational acceleration vectors during standing and sitting and then calculated the medial–lateral axis by the cross product of the two vectors. Logar and Munih [77] determined the anterior–posterior (AP) and SI axes by measuring the gravitational acceleration while standing and lying down, respectively. Four papers used the sensor orientation and segment orientation measured by an optical motion capture to obtain the sensor-to-segment matrix [34,38] or offset angles [37,68]. Studies using Xsens MVN employed a neutral pose based on the manual in MVN Studio and [62] additionally performed a T-pose and squat. This calibration procedure works based on the assumption that the segment orientation is given for each specific pose. In this regard, it is important how accurately a subject takes a specific pose [80]. Liu et al. [69] also performed standing in a neutral pose, where the segment orientations were already given. 

##### Bottom-Up Inverse Dynamics

In 24 studies, joint kinetics were estimated using bottom-up inverse dynamics, which propagates force and moment upward from the foot, the farthest extremity of the lower limb, and the external forces (i.e., GRF and GRM) required for this were measured directly through force sensors or predicted from IMU data. Faber et al. [61] and Hwang et al. [71] measured GRF from FPs instrumented on the floor to estimate the moment of the L5/S1 or lower body joint, in a lifting task and a sit-to-stand task, respectively. Eight studies measured the GRF and GRM by attaching MFPs developed or commercially available to the outsole of a shoe to estimate the joint kinetics in different motions, including walking [32,33,35,36,37,55,65,76]. Because the MFP moves with the movement of the foot, unlike FPs fixed on the floor, the GRF measured from the force sensor was transformed into a global coordinate system using the IMU attached or built into the MFP. In the other seven studies, the GRF was obtained from the pressure measured using PSs placed on the insole of the shoe. However, a PS cannot measure the horizontal component of the GRF (i.e., shear force) because it obtains the GRF from the one-dimensional pressure data. Accordingly, whereas two studies considered only the vertical component of the GRF in the inverse dynamics [64,72], five studies extended the pressure data to 3D GRF. Rouhani et al. [34,38] used a method based on the relationship between the plantar pressure distribution and the GRF components developed in a previous study [81]. Khurelbaatar et al. [40] artificially restored a 3D GRF vector that directs from the CoP to the center of gravity of the whole body. The same method was applied in later studies [78,79]. In five studies, the GRF was predicted from the IMU data rather than measured. In three of these studies, a method based on whole-body kinematics proposed by Skals et al. [82] was adopted to predict the GRF and GRM. Dorschky et al. [56] modeled foot–ground interaction based on contact dynamics [83], allowing the GRF to be determined as an optimization method. Fukutoku et al. [44] used the vertical force derived from the mass and acceleration of the segments based on the equation of motion.

##### Top-Down Inverse Dynamics

In ten papers, top-down inverse dynamics, which calculates the force and moment from the upper body segment downward, was applied as another approach. In five of these studies, inverse dynamics was performed based on the assumption that no external force was applied to the upper body segment, in order to estimate the joint moment of the lower body [68,69,74,75,77]. Among these, three studies [69,74,77] assumed that the head-arms-trunk (HAT) segments were rigid. Kodama and Watanabe [68] compared three different upper-body models segmented differently from a rigid HAT. Liu et al. [69] used the chair reaction force measured by an FP located in a chair to consider the external force generated while sitting on a chair. Yang et al. [39,41] estimated the force of the knee and ankle joints by applying top-down inverse dynamics after predicting the hip joint force using a predetermined empirical polynomial formula. Kim and Nussbaum [62] performed top-down inverse dynamics using hand force measured from the load cells attached to both sides of the box (i.e., the external force applied by the box) to estimate the shoulder moment while lifting the box. In the case of carrying the box, the moment of the L5/S1 joint was additionally estimated with the same approach. Koopman et al. [63] and Faber et al. [65] also estimated the L5/S1 joint moment while lifting a box through top-down inverse dynamics. Both studies used the hand force as an external force. In these cases, the hand force was estimated by subtracting the GRF calculated through the equation of motion (i.e., the force caused by the body mass) from the GRF (i.e., the force including the load of the box) measured from the MFP. 

##### Combined Inverse Dynamics and Others

In addition, two papers combined or compared the two approaches described above. Logar and Munih [77] estimated the moment of the lower limb joints from GRF by applying the bottom-up approach after estimating the GRF through a top-down approach. They called this the top-down-up approach. In this study, the estimation results of the top-down and top-down-up approaches were compared with those of the bottom-up approach as a reference method in an indoor experiment. Faber et al. [65] compared the results of the bottom-up method using MFP and the top-down method using hand force, which was introduced earlier. 

Zijlstra and Bisseling [73] and Kotani et al. [42] simplified the models used for estimating the hip joint moment by ignoring the GRF and force propagation to other segments. They considered the moment due to the GRF to be zero in the single stance phase of walking, assuming that the external GRF passes through the hip joint center. Thus, Zijlstra and Bisseling [73] estimated the hip adduction moment simply by taking into account only the internal components associated with accelerations, gravity, and angular accelerations. Similarly, Kotani et al. [42] estimated the hip flexion/extension moment based on the same approach (i.e., considering only the internal forces without the GRF).

#### 3.2.2. Machine Learning-Based Method (MLM)

In 15 of the selected papers, machine learning techniques were used to train models that output the joint kinetic variables by inputting IMU data based on the collected dataset. The kinetic variables for training were calculated by performing Newton–Euler-based inverse dynamics or by using dedicated software such as Visual3D (C-Motion, Germantown, MD, USA) based on the OMC and FPs. In the majority of the studies, the IMU signals for the input data were directly measured. Meanwhile, in order to deal with the issue of an insufficient amount of data, Dorschky et al. [59] and Mundt et al. [50] added the simulated IMU-like data by fusing the OMC data with the physical knowledge of biomechanics to the original IMU data.

##### Machine Learning Techniques

The most commonly used machine learning technique was neural networks, which were applied in nine studies. Among these, feedforward neural networks (FFNNs) were used in five papers [46,48,50,57,60], the convolutional neural network (CNN) was used in [59], and the remaining three papers [50,52,54] compared different techniques, including FFNN, CNN, and the long short-term memory (LSTM) network (see Table 4). Jiang et al. [45] used random forest regression to randomly select a part of the training dataset, and Matijevich et al. [67] trained the model using gradient-boosted decision trees based on an ensemble of decision trees. Refs. [49,58] trained a normalized linear regression model using the least absolute shrinkage and selection operator, often abbreviated as LASSO. Miyashita et al. [47] used multiple regression analysis and Iwama et al. [53] used linear regression analysis.

##### Input Variables

In MLMs, the model is trained based on the relationship between the joint kinetics data and the IMU signals or feature variables extracted from the signals. The input data used in each study are different. Seven papers [50,54,57,58,59,60] used triaxial accelerometer and triaxial gyroscope signals. Dorschky et al. [59] used the AP and vertical components of the accelerometer signal and the ML component of the gyroscope signal based on a 2D biomechanical model. Barua et al. [52] used the L2 norm and average extracted from the sensor signal as the feature data instead of using each axial signal, in order to be independent of the sensor attachment orientation for the convenience in practice. Iwama et al. [53] used the peak-to-peak acceleration in the ML direction based on the observation that the peak of the knee adduction moment occurs together with the peak of the ML acceleration immediately after heel contact. Lim et al. [46] and Lee et al. [48] utilized the kinematic data of the CoM because the equation of motion of the CoM simplified as a mass-spring model effectively explains the human gait. Hence, the position and velocity of the CoM were derived from the acceleration of the CoM based on mass-spring mechanics by attaching an IMU to the sacrum corresponding to the human’s CoM. Whereas most of the studies used only IMUs without any additional sensor, two studies [49,67] used both an IMU and a PS and investigated the results from various combinations of input data based on the IMU and/or PS.

### 3.3. Study Result

From the selected literature, 37 papers validated the estimation accuracy from the IMC-based wearable system by comparing the estimated kinetic variables with the truth reference values from an OMC system and the FPs. The truth reference of the joint force, moment, and power were calculated from the measured data of the reference systems by performing inverse dynamics or by using dedicated software (e.g., Visual 3D). The estimation results are listed in Table 5. Note that it is impossible to evaluate all the study results under the same conditions because the experimental protocol, attachment locations of the markers, and reference method are different for each study. The estimation accuracy was mainly evaluated using the measures indicating the correlation between the estimated value and the reference value, such as the root mean squared error (RMSE), the mean absolute error (MAE), and the correlation coefficient (CC). Some studies used normalized root mean squared error (NRMSE), which is the RMSE normalized to the parameter’s range. In addition, a number of studies used normalized values. For example, three studies [44,66,70] used the normalized force to the bodyweight (%BW), and five studies [36,43,56,59,70] used the normalized moment to the bodyweight times the body height (%BW∗BH), respectively. 

**Table 2:** Characteristics of studies.

Author (Year) [Ref.]	Activity	Joint	Kinetic Variable	Type of Method	Measurement	Subject (Number and Sex and Age)
Zijlstra and Bisseling (2004) [73]	Stance on one leg	Hip	Moment (AP)	IDM	IMU	Healthy adult (5 M, 23 (23–24))
Schepers et al. (2007) [32]	Walking	Ankle	Power, Moment (3D)	IDM	IMU, MFP	Healthy adult (1, ND)
Zheng et al. (2008) [33]	Walking	Hip, Knee, Ankle	Power, Moment (ML)	IDM	IMU, MFP	Healthy adult (8 M, 2 F, 28.1 ± 1.99)
Faber et al. (2010) [61]	Manual lifting tasks	L5/S1, Hip, Knee	Moment (3D)	IDM	IMU, FP	Healthy adult (11 M, 27.4 ± 4.3)
Krüger et al. (2011) [76]	Snowboard	Hip, Knee, Ankle	Moment (3D)	IDM	IMU, MFP	Snowboarder (1 M, 21)
Rouhani et al. (2011) [34]	Walking	Ankle	Power, Moment (3D), Force (3D)	IDM	IMU, PS	1. Ankle OA patient (8 M, 4 F, 58 ± 13) 2. Healthy adult (3 M, 7 F, 61 ± 13)
Van den Noort et al. (2012) [35]	Walking	Knee	Moment (3D)	IDM	IMU, MFP	Knee OA patient (4 M, 16 F, 61.0 ± 8.8)
Kim and Nussbaum (2013) [62]	Manual material handling tasks	L5/S1, Shoulder, Hip, Knee	Moment (3D)	IDM	IMU, FP	Healthy adult (11 M, 3 F, 22.9 ± 4.9 (19–38))
Van den Noort et al. (2013) [36]	Walking	Knee	Moment (AP)	IDM	IMU, MFP	Knee OA patient (3 M, 11 F, 61.0 ± 9.2)
Kim and Kim (2014) [55]	Squat, Sit-to-stand, Stair ascent, Walking	Hip, Knee, Ankle	Moment (ML)	IDM	IMU, MFP	Healthy adult (1 M, ND)
Liu et al. (2014) [37]	Walking	Hip, Knee, Ankle	Moment (3D)	IDM	IMU, MFP	Healthy adult (4 M, ND)
Rouhani et al. (2014) [38]	Walking	Ankle	Power, Moment (3D), Force (3D)	IDM	IMU, PS	1. Ankle OA patient (8 M, 4 F, 58 ± 13) 2. Healthy adult (3 M, 7 F, 61 ± 13)
Yang and Mao (2014) [39]	Walking	Hip, Knee, Ankle	Force (AP, SI)	IDM	IMU	Healthy adult (3 M, 24.5 ± 0.5)
Khurelbaatar et al. (2015) [40]	Walking	Cervical, Thoracic, Lumbar, Shoulder, Elbow, Wrist, Hip, Knee, Ankle	Moment (Mag), Force (Mag)	IDM	IMU, PS	Healthy adult (5 M, 27 ± 1)
Logar and Munih (2015) [77]	Ski jumping	Hip, Knee, Ankle	Moment (ML)	IDM	IMU	1. Ski jumpers (model validation) (4, 19 ± 4) 2. Ski jumpers (outdoor validation) (6, 18.9 ± 3)
Yang and Mao (2015) [41]	Walking	Hip, Knee, Ankle	Force (3D)	IDM	IMU	Healthy adult (2 M, 24.5 ± 0.5)
Faber et al. (2016) [75]	Trunk bending	L5/S1 joint	Moment (3D)	IDM	IMU	Healthy adult (9 M, 36 ± 11)
Kodama and Watanabe (2016) [68]	Squat, Sit-to-stand	Hip, Knee, Ankle	Moment (ML)	IDM	IMU	Healthy adult (6 M, 21–23)
Lee et al. (2017) [78]	Ski	Hip, Knee, Ankle	Moment (3D), Force (3D)	IDM	IMU, PS	Ski coach (7 M, 35.3 ± 4.9)
Wu et al. (2017) [72]	Stair climbing	Hip, Knee, Ankle	Moment (ML)	IDM	IMU, PS	Healthy adult (13 M, 25)
Koopman et al. (2018) [63]	Manual lifting tasks	L5/S1 joint	Moment (3D)	IDM	IMU	Healthy adult (9 M, 8 F, 33.5 ± 12.0)
Kotani et al. (2018) [42]	Walking	Hip	Moment (ML)	IDM	IMU	Healthy adult (2 M, 2 ± 0)
Liu et al. (2018) [69]	Sit-to-stand	Hip, Knee, Ankle	Moment (ML)	IDM	IMU, FP	1. Healthy adult (5 M, 28.1 ± 6.3) 2. Limb dyskinesia patients (5 M, 29.5 ± 7.5)
Purevsuren et al. (2018) [79]	Short-track skating	Knee	Moment (3D), Force (3D)	IDM	IMU, PS	Speed skater (5 M, 3 F, 16.6 ± 2.6)
Dorschky et al. (2019) [56]	Walking, Running	Hip, Knee, Ankle	Moment (ML)	IDM	IMU	Healthy adult (10 M, 27.1 ± 2.6)
Karatsidis et al. (2019) [43]	Walking	Hip, Knee, Ankle	Moment (3D), Force (3D)	IDM	IMU	Healthy adult (11 M, 31.0 ± 7.2)
Konrath et al. (2019) [70]	Stair ascent, descent and Sit-to-stand	Knee	Moment (AP), Force (SI)	IDM	IMU	Healthy adult (6 M, 2 F, 59 ± 8)
Conforti et al. (2020) [64]	Manual lifting tasks	L5/S1 joint	Force (3D)	IDM	IMU, PS	Healthy adult (1 M, 36)
Faber et al. (2020) [65]	Manual material handling tasks	L5/S1 joint	Moment (3D)	IDM	IMU, MFP	Healthy adult (8 M, 8 F, 32 ± 10)
Fukutoku et al. (2020) [44]	Walking	Knee, Ankle	Moment (ML)	IDM	IMU	Healthy adult (1 F, 24)
Larsen et al. (2020) [66]	Manual material handling tasks	L4-L5 joint	Force (3D)	IDM	IMU	Healthy adult (9 M, 4 F, 25.7 ± 3.4)
Noamani et al. (2020) [74]	Standing	L5/S1, Hip, Ankle	Moment (ML)	IDM	IMU	Healthy adult (10 M, 24.8 ± 2.8)
Hwang et al. (2021) [71]	Sit-to-stand with different weight-bearings	Hip, Knee, Ankle	Moment (ML)	IDM	IMU, FP	Healthy adult (8 M, 8 F, 27.6 ± 2.9)
Jiang et al. (2019) [45]	Walking	Ankle	Power	MLM	IMU	Healthy adult (9 M, ND)
Lim et al. (2019) [46]	Walking	Hip, Knee, Ankle	Moment (ML)	MLM	IMU	Healthy adult (7 M, 25.0 ± 2.9)
Miyashita et al. (2019) [47]	Walking	Ankle	Power	MLM	IMU	Healthy adult (13 M, 24.3 ± 5.5)
Stetter et al. (2019) [57]	16 types of movement tasks (e.g., walking, running)	Knee	Force (3D)	MLM	IMU	Sport student (13 M, 26.1 ± 2.9)
De Brabandere et al. (2020) [58]	9 types of movement tasks (e.g., walking, standing/squat on one leg)	Hip, Knee	Impulse	MLM	IMU	Hip OA patient (20, 55–75)
Dorschky et al. (2020) [59]	Walking, Running	Hip, Knee, Ankle	Moment (ML)	MLM	IMU	Healthy adult (10 M, 27.1 ± 2.6)
Lee and Park (2020) [48]	Walking	Hip, Knee, Ankle	Moment (3D)	MLM	IMU	Healthy adult (8 M, 12 F, 24.7 ± 3.2)
Matijevich et al. (2020) [49]	Running	Ankle (Tibia)	Compressive force	MLM	IMU, PS	Recreational runner (5 M, 5 F, 24 ± 2.5)
Mundt et al. (2020) [50]	Walking	Hip, Knee, Ankle	Moment (3D)	MLM	IMU	Healthy adult (ND, ND)
Mundt et al. (2020) [51]	Walking	Hip, Knee, Ankle	Moment (3D)	MLM	IMU	Healthy adult (18 M, 12 F, 28.1 ± 6.0)
Stetter et al. (2020) [60]	Walking, Running	Knee	Moment (3D)	MLM	IMU	Sport student (13 M, 26.1 ± 2.9)
Barua et al. (2021) [52]	Walking	Ankle	Power	MLM	IMU	Healthy adult (9 M, 27.1 ± 2.6)
Iwama et al. (2021) [53]	Walking	Knee	Moment (AP)	MLM	IMU	Knee OA patient (3 M, 19 F, 68.5 ± 6.4)
Matijevich et al. (2021) [67]	Manual material handling tasks	Lumbar	Moment (ML)	MLM	IMU, PS	Healthy adult (7 M, 3 F, 25 ± 3)
Mundt et al. (2021) [54]	Walking	Hip, Knee, Ankle	Moment (3D)	MLM	IMU	Healthy adult (68 M, 48 F, 37.6 ± 17.1)

Abbreviations: ML = medio-lateral; AP = anterior–posterior; SI = superior–inferior; ML and AP components of the moment are flexion/extension and ab/adduction moments, respectively; Mag = magnitude; IDM = inverse dynamics-based method; MLM = machine learning-based method; FP = force plate; MFP = mobile force plate; PS = pressure sensor; M = male; F = female; ND = not described; OA = osteoarthritis; subject’s age is stated by individual age, mean ± SD, or range (min-max).

**Table 3:** Summary of inverse dynamics-based methods.

Author (Year) [Ref.]	IMU Attachment Location	No.	GRF, Sensor or Method	Method for Joint Kinetics	Dim.	Assumption or Feature
Zijlstra and Bisseling (2004) [73]	Thorax, Pelvis	2	NA, NA	ID (Hof, 1992)	3D	Compare rigid/segmented trunk models
Schepers et al. (2007) [32]	Forefoot, Heel	2	Measured, MFP	Bottom-up ID	3D	NA
Zheng et al. (2008) [33]	Thigh (L), Calf (L), Foot (L)	3	Measured, MFP	Bottom-up ID (Hof, 1992)	2D	NA
Faber et al. (2010) [61]	Pelvis, Thigh (L), Calf (L), Foot (L)	4	Measured, FP	Bottom-up ID	3D	Simulated sensor from marker cluster
Krüger et al. (2011) [76]	Head, Sternum, Pelvis, Shoulder (R/L), Upper arms (R/L), Forearm (R/L), Hand (R/L), Thigh (R/L), Shank (R/L), Foot (R/L)	17	Measured, MFP	Bottom-up ID (in OpenSim)	3D	Multi-segment model in OpenSim (Delp, 2011)
Rouhani et al. (2011) [34]	Shank (L), Foot (L)	2	Measured, PS	Bottom-up ID	3D	1. Assuming CoP as foot’s CoR 2. Rigid foot model 3. Ignore inertial term
Van den Noort et al. (2012) [35]	Thigh (L), Shank (L), Heel (L), Forefoot (L)	4	Measured, MFP	Bottom-up ID (Hof, 1992)	3D	1. Simulated sensor from marker cluster 2. Product of GRF and moment arm only
Kim and Nussbaum (2013) [62]	Head, Sternum, Pelvis, Shoulder (R/L), Upper arms (R/L), Forearm (R/L), Hand (R/L), Thigh (R/L), Shank (R/L), Foot (R/L)	17	Lower limb: Measured, FP Shoulder: Measured (HF), Load cell	Lower limb: Bottom-up ID Shoulder: Top-down ID	3D	NA
Van den Noort et al. (2013) [36]	Shank (R/L), Heel (R/L), Forefoot (R/L)	6	Measured, MFP	Bottom-up ID (Hof, 1992)	3D	Product of GRF and moment arm only
Kim and Kim (2014) [55]	ASIS (L), Lateral femoral epicondyle (L), Lateral malleolus (L), 5th metatarsal head (L)	4	Measured, MFP	Bottom-up ID	2D	Segments move in the sagittal plane
Liu et al. (2014) [37]	Thigh (R/L), Shank (R/L), Heel (R/L), Forefeet (R/L)	8	Measured, MFP	Bottom-up ID	3D	NA
Rouhani et al. (2014) [38]	Shank (L), Hindfoot (L), Forefoot (L), Toe (L)	4	Measured, PS	Bottom-up ID	3D	1. Assuming CoP as foot’s CoR 2. 3-segment foot model
Yang and Mao (2014) [39]	GYRO: Thigh (R/L), Shank (R/L), Foot (R/L) ACC: Foot (R/L)	6	NA, NA	Lower limb: Top-down ID Hip force: 6-order polynomial function	2D	Segments move in the sagittal plane
Khurelbaatar et al. (2015) [40]	Head, Sternum, Pelvis, Shoulder (R/L), Upper arms (R/L), Forearm (R/L), Hand (R/L), Thigh (R/L), Shank (R/L), Foot (R/L)	17	Measured, PS	Bottom-up ID	3D	Restore 3D GRF from pressure data
Logar and Munih (2015) [77]	Both sides of sacrum, Upper arm (R/L), Thigh (R/L), Shank (R/L), Ski (R/L)	10	NA, NA	A1: Bottom-up ID (reference) A2: Top-down ID A3: Top-down-up ID	2D	1. Bilaterally symmetric 2. No external force on top segment
Yang and Mao (2015) [41]	GYRO: Trunk, Thigh (R/L), Shank (R/L), Foot (R/L) ACC: Foot (R/L)	7	NA, NA	Lower limb: Top-down ID Hip force: Exponential transfer function	3D	NA
Faber et al. (2016) [75]	Head, Sternum, Pelvis, Shoulder (R/L), Upper arms (R/L), Forearm (R/L), Hand (R/L), Thigh (R/L), Shank (R/L), Foot (R/L)	17	NA, NA	Top-down ID	3D	No external force on top segment
Kodama and Watanabe (2016) [68]	Upper/middle/lower trunk, Frontal/lateral side of shank/thigh (L)	7	NA, NA	Top-down ID	2D	1. Foot fixed to the ground 2. No external force on top segment 3. Compare different three trunk models
Lee et al. (2017) [78]	Head, Sternum, Pelvis, Shoulder (R/L), Upper arms (R/L), Forearm (R/L), Hand (R/L), Thigh (R/L), Shank (R/L), Foot (R/L)	17	Measured, PS	Bottom-up ID	3D	Restore 3D GRF from pressure data
Wu et al. (2017) [72]	Pelvis, Thigh (R/L), Shank (R/L), Forefoot (R/L)	7	Measured, PS	Bottom-up ID	2D	1. Segments move in the sagittal plane 2. Vertical GRF only
Koopman et al. (2018) [63]	Head, Sternum, Pelvis, Shoulder (R/L), Upper arms (R/L), Forearm (R/L), Hand (R/L), Thigh (R/L), Shank (R/L), Foot (R/L)	17	Lower limb: NA, NA Hand: Measured, FP	Lower limb: Top-down ID Hand: Bottom-up ID	3D	1. External forces only on hands 2. Compare different sensor sets (17/8/6/4 sensors)
Kotani et al. (2018) [42]	Head, upper/lower body trunk, hip (L), thigh (L), lower leg (L)	7	NA, NA	Force balance equation	2D	Consider only one-leg support
Liu et al. (2018) [69]	Trunk, Thigh (R), Shank (R)	3	Measured (CRF), FP	Top-down ID	2D	Segments move in the sagittal plane
Purevsuren et al. (2018) [79]	Head, Sternum, Pelvis, Shoulder (R/L), Upper arms (R/L), Forearm (R/L), Hand (R/L), Thigh (R/L), Shank (R/L), Foot (R/L)	17	Measured, PS	Bottom-up ID	3D	Restore 3D GRF from pressure data
Dorschky et al. (2019) [56]	Lower back, Lateral thigh (R/L), Lateral shank (R/L), Upper midfoot (R/L)	7	Predicted, Contact model	Bottom-up ID, Optimal control method (Van den Bogert, 2011)	2D	1. Construct planar MSK model 2. Compare virtual/actual sensor
Karatsidis et al. (2019) [43]	Head, Sternum, Pelvis, Shoulder (R/L), Upper arms (R/L), Forearm (R/L), Hand (R/L), Thigh (R/L), Shank (R/L), Foot (R/L)	17	Predicted, Method by Skals et al. (2017)	Bottom-up ID, Static optimization	3D	Construct MSK model (in AnyBody)
Konrath et al. (2019) [70]	Head, Sternum, Pelvis, Shoulder (R/L), Upper arms (R/L), Forearm (R/L), Hand (R/L), Thigh (R/L), Shank (R/L), Foot (R/L)	17	Predicted, Method by Skals et al. (2017)	Bottom-up ID	3D	Construct MSK model (in AnyBody)
Conforti et al. (2020) [64]	Trunk, Arm (R/L), Forearm (R/L), Pelvis, Thigh (R/L), Shank (R/L), Foot (R/L)	12	Measured, PS	Bottom-up ID	3D	1. Vertical GRF only 2. Ignore inertial forces
Faber et al. (2020) [65]	Head, Sternum, Pelvis, Shoulder (R/L), Upper arms (R/L), Forearm (R/L), Hand (R/L), Thigh (R/L), Shank (R/L), Foot (R/L)	17	Measured, MFP	A1: Bottom-up ID A2: Top-down ID	3D	External forces only on hands
Fukutoku et al. (2020) [44]	Upper body, Thigh (R/L), Lower leg (R/L), Foot (R/L)	7	Predicted, Equation of motion	Bottom-up ID	2D	1. Segments move in the sagittal plane 2. Vertical GRF only 3. Separate GRF during double support phase using zero moment point
Larsen et al. (2020) [66]	Head, Sternum, Pelvis, Shoulder (R/L), Upper arms (R/L), Forearm (R/L), Hand (R/L), Thigh (R/L), Shank (R/L), Foot (R/L)	17	Predicted, Method by Skals et al. (2017)	Bottom-up ID	3D	Construct MSK model (in AnyBody)
Noamani et al. (2020) [74]	Sternum, Sacrum (R), Tibia (R), Foot (R)	4	NA, NA	Top-down ID	3D	1. No external force on top segment 2. Bilaterally symmetric 3. Foot fixed to the ground
Hwang et al. (2021) [71]	Shank (R/L)	2	Measured, FP	Bottom-up ID	2D	1. Foot fixed to the ground 2. Segments move in the sagittal plane 3. Negligible angular/linear acceleration

Abbreviations: R = right; L = left; FP = force plate; MFP = mobile force plate; PS = pressure sensor; NA = not applicable; ID = inverse dynamics; CoR = center of rotation; GRF = ground reaction force; MSK = musculoskeletal.

**Table 4:** Summary of machine learning-based methods.

Author (Year) [Ref.]	IMU Attachment Location	No.	Technique	Input Data	Input Dim.
Jiang et al. (2019) [45]	Shank (L), Foot (L)	2	Random forests regression	2∗ACC (3D), 2*GYRO (3D)	12
Lim et al. (2019) [46]	Sacrum	1	Feedforward neural network	Time, CoM Pos/Vel/Acc (AP, V)	7
Miyashita et al. (2019) [47]	Shank (R)	1	Stepwise multiple regression	ACC (V), BW	2
Stetter et al. (2019) [57]	Thigh (R), Shank (R)	2	Feedforward neural network	2∗ACC (3D), 2*GYRO (3D)	12
De Brabandere et al. (2020) [58]	Hip (L)	1	Regularized linear regression models	ACC (3D), GYRO (3D)	6
Dorschky et al. (2020) [59]	Lower back, Thigh (R), Shank (R), Foot (R)	4	Convolutional neural network	4∗ACC (AP and V) 4∗GYRO (ML)	12
Lee and Park (2020) [48]	Sacrum	1	Feedforward neural network	time, CoM Pos/Vel/Acc (3D)	10
Matijevich et al. (2020) [49]	Shank, Foot	2	Regularized linear regression models	Different combinations of sensor data (Max/Min of shank/foot angles at midstance (IMU), features from GRF/CoP (PS), speed, slope)	
Mundt et al. (2020) [50]	ND	ND	A1. Feedforward neural network A2. Long short-term memory	ND	ND
Mundt et al. (2020) [51]	Pelvis, Thigh (R/L), Shank (R/L)	5	Feedforward neural network	5∗ACC (3D), 5*GYRO (3D)	30
Stetter et al. (2020) [60]	Thigh (R), Shank (R)	2	Feedforward neural network	2∗ACC (3D), 2*GYRO (3D)	12
Barua et al. (2021) [52]	Shank (L), Foot (L)	2	A1. Long short-term memory (LSTM) A2. Convolutional neural network (CNN) A3. Fusion of CNN and LSTM A4. Random forest regression [45]	2∗ACC Norm/Avg 2∗GYRO Norm/Avg	8
Iwama et al. (2021) [53]	Sternum, Pelvis, Thigh (R/L), Shank (R/L)	6	Linear regression	Peak-to-peak acceleration of each IMU	1
Matijevich et al. (2021) [67]	Trunk, Pelvis, Thigh (R/L), Shank (R/L), Foot (R/L)	8	Gradient boosted decision trees	Different combinations of sensor data (Kinematic data from 8 IMUs, GRF/CoP from PS)	
Mundt et al. (2021) [54]	Pelvis, Thigh (R/L), Shank (R/L)	5	A1. Multilayer perceptron A2. Long short-term memory A3. Convolutional neural network	5∗ACC (3D), 5∗GYRO (3D)	30

Abbreviations: R = right; L = left; ACC = accelerometer signal; GYRO = gyroscope signal; CoM = center of mass; ML = medial–lateral; AP = anterior–posterior; V = vertical; Pos = position; Vel = velocity; Acc = acceleration; BW = body weight; GRF = ground reaction force; CoP = center of pressure; PS = pressure sensor.

**Table 5:** Study results.

Author (Year) [Ref.]	Outcomes [Activities]	Measure	Unit	Accuracy
Zijlstra and Bisseling (2004) [73]	Hip moment (AP) [stance on one leg]	RMSE	Nm/kg	A1 (Rigid trunk model): 0.0244–0.0730 A2 (Segmented trunk model): 0.0247–0.0449
Schepers et al. (2007) [32]	Ankle power, moment (3D) [walking]	RMSE (% of peak)	Moment: Nm/N (%) Power: W/N (%)	Moment: 0.004 (2.3) Power: 0.02 (14)
Zheng et al. (2008) [33]	Hip, Knee, Ankle power, moment (ML) [walking]	RMSE (% of peak)	Moment: Nm (%) Power: W (%)	Moment: Hip = 11.2 (6.1), Knee = 7.2 (6.0), Ankle = 2.0 (5.4) Power: Hip = 5.7 (6.4), Knee = 5.7 (4.1), Ankle = 4.2 (8.4)
Faber et al. (2010) [61]	L5/S1, Hip, Knee moment (3D) [manual lifting tasks]	MAE	Nm	(L5/S1) ML = 11.5–31.0 (Hip) AP = 2.4–17.5, ML = 5.6–15.5, SI = 3.3–4.7 (Knee) AP = 1.2–3.0, ML = 1.2–2.1, SI = 0.2–4.3
Rouhani et al. (2011) [34]	Ankle power, moment (3D), force (3D) [walking]	NRMSE (CC)	% ( )	Force: AP < 9.1 (>0.97), ML < 11.5 (>0.94), SI < 3.8 (>0.91) Moment: AP < 194.0 (>0.06), ML < 13.0 (>0.99), SI < 22.7 (>0.92) Power: < 20.4 (>0.85)
Van den Noort et al. (2012) [35]	Knee moment (3D) [walking]	RMSE (% of range)	%BW∗BH (%)	AP = 0.58 (16), ML = 1.07 (26), SI = 0.10 (17)
Kim and Nussbaum (2013) [62]	L5/S1, Shoulder, Hip, Knee moment (3D) [manual material handling tasks]	MAE	Nm	(L5/S1) AP = 5.8–34.2, ML = 7.2–20.0, SI = 1.2–10.3 (Shoulder) AP = 1.0–1.5, ML = 0.6–2.2, SI = 5.8–9.9 (Hip) AP = 10.6–14.4, ML = 5.8–9.9, SI = 2.9–6.1 (Knee) ML = 5.6–6.6
Van den Noort et al. (2013) [36]	Knee moment (AP) [walking]	RMSE (% of range)	%BW∗BH (%)	0.79 (23)
Kim and Kim (2014) [55]	Hip, Knee, Ankle moment (ML) [squat, sit-to-stand, walking, etc.]	RMSE	Nm	(Hip) 8.5, (Knee) 6.5, (Ankle) 6.2
Liu et al. (2014) [37]	Hip, Knee, Ankle moment (3D) [walking]	NRMSE (CC)	% ( )	(Hip) AP = 15.3 (0.81), ML = 21.0 (0.91), SI = 19.3 (0.89) (Knee) AP = 13.4 (0.98), ML = 4.1 (0.99), SI = 9.5 (0.96) (Ankle) AP = 6.7 (0.99), ML = 3.5 (0.97), SI = 7.1 (0.95)
Khurelbaatar et al. (2015) [40]	Whole body joint moment (Mag), force (Mag) [walking]	NRMSE (CC)	% ( )	Force: 5.5–6.2 (0.71–0.99) Moment: 8.0–16.9 (0.70–0.98)
Logar and Munih (2015) [77]	Hip, Knee, Ankle moment (ML) [ski jumping]	RMSE	Nm	(Hip) 10.9, (Knee) 9.1, (Ankle) 7.5
Faber et al. (2016) [75]	L5/S1 moment (3D) [trunk bending]	RMSE (% of peak)	Nm (%)	<10 (5) (all results: graph only)
Kodama and Watanabe (2016) [68]	Hip, Knee, Ankle moment (ML) [squat, sit-to-stand]	RMSE (CC)	Nm/kg ( )	Avg: 0.06 (Hip, Knee = 0.98, Ankle = 0.80) (all results: graph only)
Koopman et al. (2018) [63]	L5/S1 moment (3D) [manual lifting tasks]	RMSE	Nm	Set A (17 sensors, i.e., full body): 16.6 Set B (8 sensors): 20.5 Set C (6 sensors): 22.0 Set D (4 sensors): 30.6
Dorschky et al. (2019) [56]	Hip, Knee, Ankle moment (ML) [walking, running]	RMSE (CC)	%BW∗BH	(Hip) 1.5–3.2 (0.76–0.85) (Knee) 1.5–3.4 (0.81–0.94) (Ankle) 1.6–3.2 (0.95–0.96)
Karatsidis et al. (2019) [43]	Hip, Knee, Ankle moment (3D), force (3D) [walking]	RMSE (CC)	Force: %BW ( ) Moment: %BW∗BH ( )	Force: (Hip) AP = 17.6 (0.71), ML = 27.0 (0.73), SI = 102.8 (0.78) (Knee) AP = 30.6 (0.82), ML = 12.0 (0.91), SI = 63.1 (0.90) (Ankle) AP = 22.2 (0.84), ML = 24.3 (0.93), SI = 88.5 (0.93) Moment: (Hip) AP = 1.4 (0.83), ML = 2.2 (0.92), SI = 0.5 (0.50) (Knee) AP = 1.1 (0.81), ML = 1.9 (0.58), SI = 0.3 (0.73) (Ankle) AP = 0.6 (0.76), ML = 1.6 (0.93), SI = 0.5 (0.67)
Konrath et al. (2019) [70]	Knee moment (AP), Force (SI) [stair ascent/descent, sit-to-stand]	RMSE (CC)	Force: %BW ( ) Moment: %BW∗BH ( )	Force: 40–90 (0.85–0.92) Moment: 0.6–1.4 (0.74–0.98)
Conforti et al. (2020) [64]	L5/S1 force peak (3D) [manual lifting tasks]	MAE	N	AP = 11.7–12.8, ML = 4.5–5.8, SI = 11.7–20.9
Faber et al. (2020) [65]	L5/S1 moment (3D) [manual material handling tasks]	RMSE (% of peak)	Nm (%)	A1 (bottom-up) < 40 (20%) A2 (top-down) < 20 (10%) (all results: graph only)
Larsen et al. (2020) [66]	L4-L5 joint force (3D) [manual material handling tasks]	RMSE	%BW	AP = 7.98–22.73 ML = 1.71–4.06 SI = 44.87–74.69
Noamani et al. (2020) [74]	L5/S1, Hip, Ankle moment (ML) [standing]	RMSE (CC)	Nm/kg ( )	<0.016 (>0.93) (all results: graph only)
Hwang et al. (2021) [71]	Hip, Knee, Ankle moment (ML) [sit-to-stand]	RMSE (CC)	Nm/kg ( )	(Hip) 0.044–0.105 (0.987–0.995) (Knee) 0.041–0.091 (0.990–0.999) (Ankle) 0.024–0.028 (0.988–0.996)
Jiang et al. (2019) [45]	Ankle power [walking]	NRMSE (CC)	W/kg ( )	Intra-subject test: 0.03–0.10 (0.94–0.98) Inter-subject test: 0.06–0.21 (0.84–0.93)
Lim et al. (2019) [46]	Hip, Knee, Ankle moment (ML) [walking]	NRMSE	%	Hip = 10.65–11.67 Knee = 9.33–10.58 Ankle = 9.24–9.64
Stetter et al. (2019) [57]	Knee force (3D) [16 types of movement tasks]	CC		AP = 0.64–0.90 ML = 0.25–0.60 SI = 0.60–0.94
De Brabandere et al. (2020) [58]	Hip, knee impulse [9 types of movement tasks]	MAPE	%	(Hip) R = 36, L = 29 (Knee) R = 48.2, L = 32.1
Dorschky et al. (2020) [59]	Hip, Knee, Ankle moment (ML) [walking, running]	RMSE (CC)	%BW∗BH ( )	(Hip) < 1.78 (>0.927) (Knee) < 1.28 (>0.958) (Ankle) < 1.39 (>0.971)
Lee and Park (2020) [48]	Hip, Knee, Ankle moment (3D) [walking]	NRMSE	%	(Hip) AP = 15.38–22.50, ML = 9.08–16.08, SI = 13.72–23.66 (Knee) AP = 15.95–20.96, ML = 17.47–33.64, SI = 16.16–27.62 (Ankle) ML = 11.54–18.20
Matijevich et al. (2020) [49]	Tibial compressive force [running]	NRMSE	%	A1 (IDM): 5.2 A2 (MLM using PS and foot/shank IMU): 2.6 A3 (MLM using PS): 4.7 A4 (MLM using foot/shank IMU): 8.3
Mundt et al. (2020) [50]	Hip, Knee, Ankle moment (3D) [walking]	NRMSE (CC)	% ( )	(Hip) A1 (FFNN): AP = 7.34 (0.99), ML = 10.29 (0.98), SI = 6.50 (0.99) A2 (LSTM): AP = 8.34 (0.98), ML = 9.83 (0.99), SI = 8.64 (0.99) (Knee) A1: AP = 10.58 (0.98), ML = 9.46 (0.98), SI = 17.12 (0.88) A2: AP = 14.52 (0.96), ML = 11.85 (0.96), SI = 20.05 (0.86) (Ankle) A1: AP = 22.60 (0.91), ML = 7.36 (0.99), SI = 17.59 (0.93) A2: AP = 24.19 (0.90), ML = 7.32 (0.99), SI = 19.68 (0.94)
Mundt et al. (2020) [51]	Hip, Knee, Ankle moment (3D) [walking]	NRMSE (CC)	% ( )	<13.0 (Avg = 0.95) (all results: graph only)
Stetter et al. (2020) [60]	Knee moment (3D) [6 types of movement tasks]	RMSE (CC)	Nm/kg ( )	AP = 0.18–0.92 (−0.05–0.71) ML = 0.26–1.13 (0.65–0.85)
Barua et al. (2021) [52]	Ankle power [walking]	MSE (CC)	ND ( )	A1 (LSTM) = 0.059 (92.69) A2 (CNN) = 0.127 (92.27) A3 (CNN-LSTM) = 0.129 (92.07) A4 (Random Forest) = 0.184 (81.75)
Iwama et al. (2021) [53]	Knee moment (AP) [walking]	RMSE (*p*-value)	Nm/(kgm) ( )	0.079–0.084 (< 0.001)
Matijevich et al. (2021) [67]	Lumbar moment (ML) [manual material handling tasks]	RMSE	Nm	Set A (Trunk IMU) = 31 Set B (Trunk IMU + PS) = 20 Set C (Distributed sensors) = 17
Mundt et al. (2021) [54]	Hip, Knee, Ankle moment (3D) [walking]	NRMSE	%	(all results: graph only)

Abbreviations: AP = anterior–posterior; ML = medial–lateral; SI = superior–inferior; CC = correlation coefficient; RMSE = root mean squared error; MAE = maximum absolute error; NRMSE = normalized root mean squared error; Mag = magnitude; Avg = average; BW = body weight; H = height; FFNN = feedforward neural network; LSTM = long short-term memory.

Some studies investigated the accuracy difference due to different estimation factors, such as biomechanical modeling and types of neural networks. Refs. [68,73] compared the joint moment estimated using the rigid trunk model, which considers the head, arms, and trunk (HAT) to be one rigid segment, and the segmented trunk model. As a result, the segmented trunk model showed a higher accuracy than the rigid trunk model. Koopman et al. [63] compared the four sensor sets (17/8/6/4 sensors) in estimating the L5/S1 moment during lifting, and the full set of the 17 sensors showed a lower error (16.6 Nm) compared to the set with fewer sensors. Faber et al. [65] compared the L5/S1 moment estimated through the bottom-up and top-down models based on full-body sensor sets, and the latter model showed about half the RMSE compared to the former model (bottom-up <40 Nm, top-down <20 Nm). In addition, Logar and Munih [77] compared the top-down and top-down-up approaches, where the latter approach showed a higher accuracy. Matijevich et al. [49] compared the accuracies of peak tibial force estimated through IDM and MLM. In the latter, various combinations of input data based on IMUs and PS were tried. As a result, the case of using both IMU and PS data for machine learning showed the highest accuracy, which was superior to IDM (NRMSE: IDM = 5.2%, MLM = 2.6%). In three studies [50,52,54], the estimation performances of different neural networks were compared. In [54], three neural networks (multilayer perceptron, CNN, and LSTM) for estimating the 3D joint kinetics of the lower body were compared. As a result, CNN achieved the highest performance among the three neural networks compared, but it was disadvantageous for real-time estimation due to the large datasets and the elaborate preprocessing requirement. In summary, in IDM, a detailed model, a large number of sensors, and an appropriate approach contributed to the estimation accuracy, and in MLM, the selection of input variables and machine learning techniques affected the accuracy. 

## 4. Discussion

In this paper, we explored the methodologies of the current studies on estimating the joint kinetic variables by means of an IMC-based wearable system. This paper focused on the characteristics (Table 2), the methodologies (Table 3 for IDM and Table 4 for MLM), and the results (Table 5) of the selected studies.

### 4.1. Characteristics of Studies

The study characteristics described in Section 3.1 indicated that IMC technologies were utilized to evaluate joint kinetics for a variety of activities and subjects in the selected papers. The activities analyzed ranged from daily activities, such as walking or stair ascent, to sports activities. For example, four studies [76,77,78,79] estimated joint moments during winter sports such as skiing and skating, which are difficult to evaluate with laboratory-based measurement systems, owing to the vast range of activities. This shows the potential of the IMC system to be applied to motion analysis for outside-lab environments. Because numerous movements in daily life, such as walking, are driven by the control of the lower body joints, the majority of the studies focused on the joint kinetics of the lower extremities. Regarding the kinetic variables for evaluating the load on the joint, the joint force and moment were estimated the most frequently. Moreover, because the joint contact force is determined by adding the joint reaction and muscular forces, and the joint power is calculated by multiplying the joint moment and angular velocity of the body segment, joint force and moment are important in determining these variables. In addition, most of the studies evaluated kinetics in healthy subjects, and in only seven papers were patients included in the subject group. This indicates that the majority of the studies focused on evaluating the estimation accuracy of the kinetic variables rather than investigating specific hypotheses with respect to joint function and health.

### 4.2. Inverse Dynamics-Based Method and Its Limitations

In IDM, inverse dynamics is performed based on a biomechanical modeling through the IMC system, which is divided into the bottom-up and top-down approaches. A bottom-up approach estimates the joint force and moment upward, using an external force, i.e., GRF, caused by contact between the feet and the ground. The GRF is measured directly using force sensors (e.g., MFP and PS) or predicted from the IMU data. In the former case, because the GRF was directly measured using a force sensor, the uncertainty factor was relatively small, whereas the latter case had a greater uncertainty owing to the prediction error of the GRF. Nevertheless, the latter case was meaningful in that it used only IMUs, without any additional sensors which may hinder wearability. In studies performing top-down inverse dynamics, it was assumed that there was no external force applied to the upper body or that the external force was applied only to the hand while lifting an object. In this case, the joint force and moment were calculated downward from the inertial force of the upper bodies or the external force applied to the hand, and the GRF applied to the foot could be estimated. Therefore, selected studies evaluated the estimation accuracy of the GRF with joint kinetics [39,41,68,74,75,77]. Furthermore, in other studies using this approach, HAT segments were assumed to be one rigid body only for activities with small upper body motion, such as sit-to-stand or squat. This considers the fact that it is difficult to measure a clear external force applied to the upper body, unlike the GRF generated by foot–ground contact. Nevertheless, an acceptable level of estimation accuracy was identified. For example, in [74], the RMSE of the estimated joint moment was less than 0.016 Nm/kg, which was similar to the accuracy of the OMC-based top-down approach. To summarize, the selection criteria for a specific IDM may be the following: (i) the existence of external forces (e.g., load-free hands versus GRF-applied feet) and (ii) how to obtain external forces, if any (e.g., direct measurement by MFP versus estimation by IMUs and modellings).

As discussed in [28], the IDM is based on a biomechanical model, and as the degree of freedom of this model increases, the number of sensors required for it also increases. For example, in 11 studies, full-body kinematic data were measured using 17 IMUs attached to segments of the entire body. These methods have the advantage of being able to directly drive a model and accurately analyze joint kinetics based on the kinematics estimated through the sensors attached to each segment. However, as a large number of sensors are attached to the bodies, it becomes cumbersome and requires the processing of a large amount of data. In this regard, recent studies that estimate kinematics using sparse sensor setup are noteworthy for their applicability in kinetics analysis [84,85]. Another inherent limitation of IDM is that the accuracy of kinematics (e.g., segment orientation and joint center position) estimated by means of an IMC system affects the estimation of joint kinetics. One of the sources of inaccurate kinematics is soft tissue artifacts. The soft tissue artifacts cause misalignment between the segment frame and the sensor frame and the estimation error of the joint center positions, leading to an inaccurate estimation of the segment coordinate system and joint moment, respectively. These issues still remain an open question in sensor-to-segment calibrations [86,87,88]. In estimating the joint center position, two methods of estimating the sensor-to-joint vector as a time-varying one have been proposed to consider soft tissue artifacts, but they have a limitation in that they only work in motions where the joint center is moveless due to kinematic constraints [89,90]. These unresolved problems related to the segment coordinate systems and the joint center positions will be one future research direction.

### 4.3. Machine Learning-Based Method and Its Limitations

The MLMs estimate joint kinetic variables without performing inverse dynamics by training a model based on a dataset consisting of the truth reference values and the IMU data. In this method, different factors, including the selection of input data and the machine learning techniques, as well as sensor attachment, contribute to the estimation accuracy. Therefore, each paper carefully considered and selected the features, machine learning architecture, and sensor attachment location. Moreover, large datasets are an important factor in terms of estimation performance. Accordingly, the majority of the studies trained models based on sufficient data, and two studies [51,59] augmented the IMU data with the simulated IMU signals from the OMC data. In most of the studies using MLM, a relatively small number of sensors was used compared to IDM. De Brabandere et al. [58] estimated the joint load only using a single sensor embedded in a smartphone. As such, MLM has the advantage of providing convenience to users as it may work for a simpler system. This benefit is critical, especially for patients with reduced mobility during rehabilitation.

The MLM indirectly estimates kinetic variables through machine learning; so, it is independent of the issues of anatomical calibration. However, as this method also uses a skin-attached sensor, the distortion due to the soft tissue artifacts (e.g., the vibration of the skin and misalignment) is unavoidable. To reduce such distortion as much as possible, the selection of an appropriate attachment location of the sensor or a firm fixation of the sensor to the segments is needed [57]. In addition, there is also a factor of uncertainty that inconsistent estimation results could be obtained depending on the physical characteristics of the subject and the environment. In the MLMs, sufficient data also need to be collected to achieve a high estimation performance. In this regard, Refs. [50,59] augmented the IMU data by generating accelerometer and gyroscope signals from the OMC data. However, the IMU data generated from the OMC data do not include the effects of soft tissue artifacts; so, it appears different from the actual sensor signals. Therefore, research that can overcome the limitations, with consideration of these effects, is needed.

### 4.4. Study Results

Of the selected literature, 37 validated the estimation accuracy of the developed system. They evaluated the accuracy of the estimated variables using the truth reference values determined by means of an OMC and FPs. In the case of walking, analyzed in many studies, the estimation results of lower limb joint kinetics could be achieved with an excellently high correlation with the truth reference. For example, Refs. [37,51] showed a CC close to or higher than 0.9 for the 3D moments of the three joints. It was also confirmed that for other activities, such as material handling tasks or sit-to-stand, the IMC-based wearable system produced estimated results similar to those of the laboratory-based system. For the lumbar moment during the material handling tasks, an MLM that used trunk IMU and PS in [67] achieved an RMSE of 20 Nm, almost equivalent to that of [65] (top-down inverse dynamics based on full-body kinematics). Some papers reported a wide range of estimation accuracy for a variety of movement tasks [57,60,62]. Ref. [57] reported results for a total of 16 movement tasks, including walking/running in various conditions, and the relative root mean square error (rRMSE) of knee vertical force ranged from 14.2 to 25.9%. Among the 16 tasks, the sprint start and two-leg jump landing yielded the highest rRMSE (25.9% each), while walking and moderate running yielded the lowest rRMSE (14.2% each). In addition, some studies suggested the possibility of joint kinetics analysis in outdoor exercise such as winter sports. In Refs. [78,79], joint kinetics during speed skating and skiing were estimated using the wearable system developed in [40], respectively, but the estimation accuracy was not evaluated in both papers. However, the developed system of [40] showed a high correlation with the truth reference (CC of 0.96 or higher), which shows some possibility that it can be applied to winter sports.

Overall, many results have been reported on walking (37 papers), manual lifting/handling tasks (7 papers), sit-to-stand (5 papers), and running (5 papers). These results may be helpful to refer to for evaluating joint kinetics for the above activities in future studies. However, it should be noted that each result was measured under different conditions, such as different experimental protocols, the biomechanical model, or evaluation metrics. In particular, the choice of the model, including the segment coordinate system and the joint parameters in the OMC system, may affect the results of joint kinetics [91,92]. In this regard, careful attention should be taken in the comparison and interpretation of the results of each paper. In addition, even though the OMC system is used as a validation system, it has inaccuracies in that it indirectly measures the joint kinetics. The OMC system uses skin-attached reflective markers, and thus, it is affected by soft tissue artifacts, leading to errors in the kinematics. However, it should be noted that this review focuses on how close to the laboratory-based systems the IMC system can produce results, rather than how accurately it can estimate variables in terms of in vivo kinetics.

## 5. Conclusions

Joint kinetic variables have been estimated by performing inverse dynamics from the measured data with a laboratory-based measurement system (i.e., OMC and FPs). However, the measurement environment is restricted because the OMC system operates in a fixed space. Conversely, a number of recent studies have developed IMC-based wearable systems with the potential to overcome these limitations and to perform the kinetic analysis of joints in different activities.

The methodologies of the studies reviewed in this paper were largely divided into the case of performing inverse dynamics based on kinematic data (IDM) and the case of using machine learning techniques (MLM) to estimate the joint kinetics. Whereas the IDM requires the appropriate selection of biomechanical modeling, the inverse dynamics approach, and a method of measuring external force, the MLM requires the appropriate selection of input data, machine learning techniques, and sensor attachment. These methods present different characteristics in estimating kinetic variables; however, it is demonstrated that both methods could be applied with good performance for kinetic analysis of joints in different daily activities such as walking and running. In future studies, it will be necessary to directly compare and analyze the estimation results of joint kinetics using these two methods for various activities. In addition, IDM has a disadvantage in that segment/joint kinematics affects the estimation accuracy of joint kinetics. From this point of view, it would be valuable to analyze the effect of the inaccuracy of IMC-based kinematics on IMC-based joint kinetics and to find a solution for this.

## Figures and Tables

**Table 1:** Search terms applied for the literature search.

Categories	Search Terms
Joint	(joint * OR limb OR ankle OR knee OR hip OR lumbar OR L5S1 OR L5/S1 OR shoulder OR elbow OR wrist OR shoulder)
	AND
Kinetics	(kinetic * OR power OR moment * OR torque * OR force * OR load *)
	AND
IMU	(“inertial sensor *” OR “inertial measurement unit *” OR “inertial motion capture” OR IMU OR MARG OR “orientation sensor *” OR “motion sensor *” OR gyroscope OR accelerometer)

Asterisks (*) were used to find words with different endings.

## Abbreviations

IMC	Inertial motion capture
OMC	Optical motion capture
IMU	Inertial measurement unit
FP	Force plate
MFP	Mobile force plate
PS	Pressure sensor
GRF	Ground reaction force
GRM	Ground reaction moment
CoP	Center of pressure
CoM	Center of mass
HAT	Head-arms-trunk
IDM	Inverse dynamics-based method
MLM	Machine learning-based method
FFNN	Feedforward neural network
CNN	Convolutional neural network
LSTM	Long short-term memory
AP	Anterior–posterior
ML	Medial–lateral
SI	Superior–inferior
